# A new immunoprecipitation-real time quantitative PCR assay for anti-Th/To and anti-U3RNP antibody detection in systemic sclerosis

**DOI:** 10.1186/ar3858

**Published:** 2012-05-29

**Authors:** Angela Ceribelli, Minoru Satoh, Edward KL Chan

**Affiliations:** 1 Department of Oral Biology, University of Florida, P.O. Box 100424, 1395 Center Drive, Gainesville, FL 32610-0424, USA; 2 Division of Rheumatology and Clinical Immunology, Department of Medicine, and Department of Pathology, Immunology, and Laboratory Medicine, University of Florida, P.O. Box 100221, 1600 SW Archer Rd, Gainesville, FL 32610-0221, USA

## Abstract

**Introduction:**

Classic anti-nucleolar antibodies anti-Th/To and U3 ribonucleoprotein (-U3RNP) can help in the diagnosis, prediction of organ involvement and prognosis in systemic sclerosis (SSc); however, no validated commercial assay is available. We aimed at establishing a novel quantitative real time PCR (qPCR) method to detect these antibodies.

**Methods:**

Standard immunoprecipitation (IP) was performed using K562 cell extract and RNA components were extracted. cDNA was reverse transcribed from RNA components and Th RNA and U3 RNA were detected by qPCR using custom primers. Cycle threshold (Ct) values were compared in a titration experiment to determine the assay efficacy. The new assay was evaluated by testing 22 anti-Th/To and 12 anti-U3RNP positive samples in addition to 88 controls, and the results were compared with IP as a gold standard.

**Results:**

By testing serial 1:8 dilutions of cell lysate as the substrate in the IP step, RNA extracted after IP, and its derived cDNA, linear dose response curves were noted for both anti-Th/To and -U3RNP. With every dilution, Ct values changed approximately three as expected, reflecting the eight-fold difference of cDNA. The Ct difference between positive and negative samples was 8 to 13, which was similar throughout the dilutions. In the specificity analysis, the Ct values of positive samples were clearly different from the negative groups and the results by qPCR had a near perfect correlation with IP.

**Conclusions:**

Our new method readily detects these two clinically important antibodies in SSc. Making tests for anti-Th/To and -U3RNP antibodies widely available to clinicians should be helpful in the diagnosis and follow-up of SSc patients.

## Introduction

Scleroderma (Systemic Sclerosis, SSc) is a systemic autoimmune disease characterized by fibrosis, vascular changes, and the production of autoantibodies. The most common antibodies associated with SSc are anti-centromere (ACA), -topoisomerase I (topo I) and -RNA polymerase III (RNAPIII) antibodies, approximately 20% each [1-5]. Anti-topo I and ACA have been used for about 30 years for diagnostic purposes, while anti-RNAPIII ELISA has been added to routine screening only recently [6-8]. SSc patients can be classified into two major subsets: limited (lcSSc) and diffuse (dcSSc) cutaneous variants. The dcSSc is frequently associated with anti-topo I, -RNAPIII, or -U3RNP, while lcSSc is associated with ACA and anti-Th/To antibodies [1,9]. These autoantibodies are fairly specific for SSc and can be detected even before diagnosis. They are associated with unique clinical features and are useful in predicting clinical manifestations of SSc [1,10-12].

Anti-Th/To and -U3RNP are anti-nucleolar antibodies that have been known for more than 25 years. Despite their clinical importance, these SSc autoantibodies have not been utilized clinically because of the unavailability of antibody testing [7,13]. Urea-polyacrylamide gel electrophoresis (PAGE) analysis of the RNA components in immunoprecipitates, either by silver staining or by using ^32^P-labeling of cells, is the standard method, but it is performed only in a small number of research laboratories. No commercial widely-available validated immunoassay kit has been produced so far [14]. The aim of our study is to establish a new method to detect anti-Th/To and -U3RNP antibodies based on quantitative PCR (qPCR) detection of the RNA components of the ribonucleoprotein autoantigens.

## Materials and methods

### Immunoprecipitation and quantitative PCR

Immunoprecipitation (IP) was performed using K562 cell lysate and associated RNA was extracted using phenol/chloroform/isoamyl alcohol (25:24:1) as described [13,15]. RNA pellets were resuspended in 30 µl RNA-grade water. cDNA was obtained from each RNA sample (10 µl) by reverse transcription (RT) using RT Master Mix (High Capacity cDNA RT kit, Applied Biosystems Inc., ABI, Foster City, CA, USA). The thermal cycler for the RT setting was: 10 minutes at 25°C, 120 minutes at 37°C, 5 seconds at 85°C. Quantitative PCR (qPCR) was performed using the TaqMan Fast Universal PCR Master Mix (ABI). For Th RNA (7-2 RNA, RMRP) detection, 'Hs03298751_s1' primer by ABI was used, while the primer for U3 RNA (SNORD3A, Integrated DNA Technologies, Coralville, IA, USA) has the following sequence: Probe 5'-/56-FAM/CCAAGCAAC/ZEN/GCCAGAAAGCCG/3IABkFQ/-3'; Primer 1 (FOR.) 5'-TGTAGAGCACCGAAAACCAC-3'; Primer 2 (REV.) 5'-TCCCTCTCACTCCCCAATAC-3'. qPCR was performed in duplicate using the StepOne cycler (ABI) for 40 cycles, and results were evaluated by cycle threshold (Ct) values.

In some experiments, La-depleted cell extract was also used to examine the effects of La depletion in a limited number of samples (n = 24). An extract from 25 × 10^6 ^K562 cells was absorbed with 0.5 ml of anti-La immunoglobulin G-cyanogen bromide (IgG-CNBr)-activated Sepharose 4B beads to deplete La [16].

### Serum samples

The protocol of this study was approved by the Institutional Review Board (IRB). This study meets and is in compliance with all ethical standards in medicine and informed consent was obtained from all patients according to the Declaration of Helsinki. For the titration experiment, one each of anti-Th/To, -U3RNP and normal human serum (NHS) was tested, with serial eight-fold dilutions (from 1 to 1:4096) of cell lysate as substrate in the IP step, RNA extracted after IP, and its derived cDNA. To validate the new assay, 22 anti-Th/To, 12 anti-U3RNP-positive samples, 58 SSc sera negative for anti-Th/To and -U3RNP antibodies (20 anti-Topo I, 18 anti-RNAPIII, 15 ACA, 5 anti-PM-Scl), 12 anti-La/SSB, 3 anti-trimethylguanosine (TMG) [17], and 15 NHS, were analyzed. The specificity of these sera was confirmed by IP and urea-PAGE analysis as described [13]. Our data are representative of two or more independent experiments. PCR was analyzed in duplicate and immunoprecipitation was also tested in duplicate in some experiments. The typical reproducibility was very high showing a difference within one Ct value. In the new assay described here, no housekeeping gene mRNA would be present constantly, which is different from the standard qPCR used to examine the levels of mRNA for the gene of interest in total RNA or total mRNA. Nevertheless, the level of 18S RNA was also examined as a potential internal control for nonspecific binding (data not shown), but the results showed variation in its levels so that it was not considered suitable as an internal control in our assay.

### Statistical analysis

Data between groups were compared by Kruskal-Wallis and Dunn's multiple comparison tests using Prism 5.0 for Macintosh (GraphPad Software, Inc). The same program was applied to obtain the receiver operating characteristic (ROC) curves, comparing the anti-Th/To or -U3RNP positive group versus the negative group to determine the cut-off. Statistical significance was accepted at *P *<0.05.

## Results

### Autoantibody detection using qPCR for the RNA component of the ribonucleoprotein autoantigen complex

**Figure 1:**
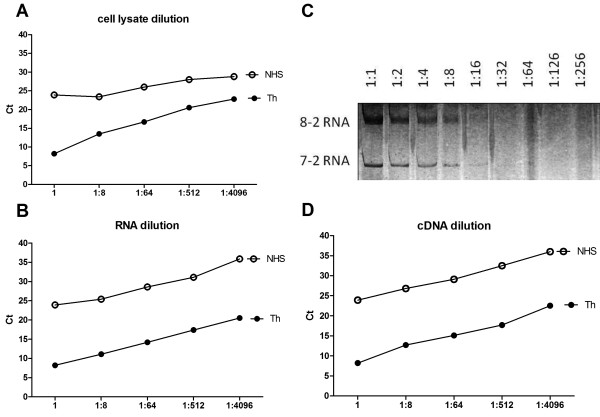
**High sensitivity and linear detection of anti-Th/To antibodies in the IP-qPCR assay demonstrated by titration analyses**. One prototype anti-Th/To sample and NHS were analyzed, and Ct values plotted against serial dilution of cell lysate used as substrate for IP (**A**), RNA extracted from IP (**B**), or the derived cDNA (**D**). Dilutions start with lysate from 10^7 ^K562 cells per sample. **C**. The sensitivity of our new IP-qPCR method is compared with RNA dilution detected by silver staining, which allows the identification of the two RNA bands (8-2 RNA, 7-2 RNA) only up to 1:16 dilution. Ct, cycle threshold; IP-qPCR, immunoprecipitation-quantitative polymerase chain reaction; NHS, normal human serum.

The sensitivity and linearity of the assay was evaluated for the detection of the Th and U3 RNA components, using serial 1:8 dilutions (1-1:4096) of cell lysate, RNA purified after IP (both started from extracts of 10^7 ^cells per sample, but data shown are based on the use of 10 out of 30 µl of RNA, equivalent to 3.3 × 10^6 ^cells) and cDNA obtained after RT (the equivalent of cDNA made from RNA derived from 3.3 × 10^6 ^cells) (Figure 1). Ct values for the titration using serially diluted cell lysate were 8.3 (extract from 10^7 ^cells, equivalent to undiluted or '1' in Figure 1A) to 22.7 (extract from 2.4 × 10^3 ^cells, corresponding to 1:4096 in Figure 1A) for Th RNA and 13.4 to 28.9 for U3 RNA (not shown), using the prototype sera. Linear dose response curves were noted for cell lysate, RNA, and cDNA dilutions, and the difference between positive samples and NHS was clear even at 1:4096 dilution, which corresponded to a cell extract from 2.4 × 10^3 ^K562 cells (Figure 1A). Ct values changed by approximately three for every 1:8 dilution, reflecting the eight-fold difference and the high efficiency of the qRT-PCR as expected (Figure 1A). Similar titration of RNA and cDNA, shown in Figure 1B and 1D, demonstrated the same linear relationship in each step of the qRT-PCR. IP using the standard 8 µl of human serum versus 1 µl was also compared in the titration experiment, to examine the effects on the Ct values. The difference in Ct values was approximately three, further demonstrating the high sensitivity of the new method also with 1 µl of serum.

The sensitivity of detection of Th or U3 RNA by the qPCR method was compared with that of the standard urea-PAGE-silver staining method by testing the same amount of serially diluted Th RNA (Figure 1B versus C) or U3 RNA (not shown). When using serial dilutions of RNA, the qPCR assay clearly detected a signal at the highest dilution of 1:4096 (Figure 1B) whereas Th RNA was barely visible only up to 1:16 dilution by silver staining (Figure 1C). Thus, qPCR was at least 256-fold more sensitive than silver staining. For U3 RNA detection, qPCR was clearly positive at a dilution of 1:4096, similarly to 7-2 RNA, whereas U3 RNA was detected only up to a 1:8 dilution by silver staining (data not shown), indicating that the qPCR assay was at least 512-fold more sensitive. Based on these data, cell extracts from one million cells, which correspond to 1/10th the amount of cell lysate normally used for IP, were used for screening of sera for anti-Th/To, -U3RNP, and other antibodies.

### Specificity of IP-qPCR assay applied to clinical samples

**Figure 2:**
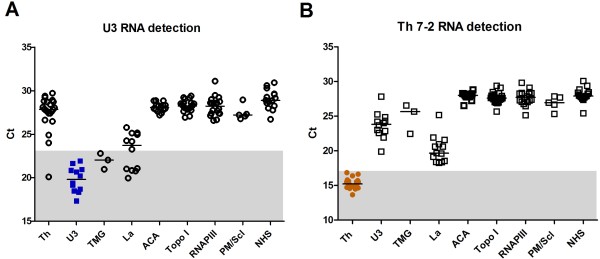
**Specific detection of anti-Th/To and U3RNP antibodies by IP-qPCR assay**. **A, B**. Sera with antibodies to Th/To (n = 22), U3RNP (n = 12), TMG (n = 3), La (n = 12), topo I (n = 20), RNAPIII (n = 18), ACA (n = 15), PM-Scl (n = 5), and normal human sera (NHS, n= 15) were tested by the IP-qPCR method. Data points and median bar are representative of at least two independent experiments. *P *< 0.05 by Kruskal-Wallis and Dunn's multiple comparison tests was present among all the groups, with the exception of the anti-U3RNP/anti-TMG groups (*P *= ns). The cut-off was determined by ROC curve and positive range is shown as shaded areas. ACA, anti-centromere antibodies; IP-qPCR, immunoprecipitation-quantitative polymerase chain reaction; NHS, normal human serum; RNAPIII, RNA polymerase III; ROC curve, receiver operating characteristic curve; TMG, trimethylguanosine; topo I, topoisomerase I.

The mean Ct value was 19.81 (standard deviation (SD) 1.45) for anti-U3RNP and 15.27 (SD 0.73) for anti-Th/To samples (Figure 2A, B). These values were clearly separated from those of the control groups, with a difference of approximately eight Ct values for anti-U3RNP and approximately 13 Ct values for anti-Th/To (*P *<0.05) (Figure 2A, B). The Ct values of anti-TMG sera were similar to those of anti-U3RNP positive sera (Figure 2A). This was expected since anti-TMG antibodies recognize the cap structure on U1-U5RNA, including U3RNA [17]. Despite maintaining statistical significance, the Ct difference between anti-Th and -La samples was smaller than the Ct difference between anti-Th and other negative controls (Figure 2C). This could be explained based on the interaction and co-immunoprecipitation of Th RNA by La antigens, as shown previously [18]. For this reason, anti-La sera were also tested in the IP-qPCR assay using La-depleted K562 cell lysate. Ct values increased by about two and became comparable to other negative groups when La-depleted cell lysate was used in place of whole cell lysate (data not shown). This indicated that the low Ct values of anti-La sera in the anti-Th qPCR assay were due to co-IP of the Th RNA by the La complex, and not by coexisting anti-Th antibodies. When testing anti-U3RNP samples, 5/12 anti-La positive samples had low Ct values, which fell below the cut-off (Figure 2A). This might be explained by the observation that La could bind and stabilize precursors of the U3 small nucleolar RNA to prevent degradation [19]. Thus, U3RNA could be weakly detected in the anti-La immunoprecipitate.

Considering IP with silver staining as the gold standard for the detection of anti-Th and -U3RNP antibodies, the sensitivity by our new IP-qPCR method was 100% for both (Table 1). Assay specificity for anti-Th and -U3RNP in SSc patients was 100% and 98.9%, respectively (Table 1). For anti-U3RNP detection, one anti-Th/To serum was repeatedly positive for an unknown reason.

**Table 1:** Sensitivity and specificity of our new IP-qPCR method.

**Autoantibody specificity**^a^	n	Anti-Th/To by IP-qPCR	Anti-U3RNP by IP-qPCR
***Scleroderma***			

Th/To	22	100%	4.5% (1/22)
U3RNP	12	0%	100%
Topo I	20	0%	0%
RNAPIII	18	0%	0%
ACA	15	0%	0%
PM-Scl	5	0%	0%

***Others ***			

TMG	3	0%	100%
La	12	0%	42% (5/12)
NHS	15	0%	0%

Results show the high sensitivity and specificity of the new assay, for the detection of anti-Th/To and -U3RNP antibodies, compared with silver staining as gold standard. ^a^Antibody specificity validated by IP, urea-PAGE, and silver staining. ACA, anti-centromere antibodies; IP, immunoprecipitation; NHS, normal human serum; RNAPIII, RNA polymerase III; TMG, trimethylguanosine; topo I, topoisomerase I.

## Discussion

The importance of the development of a convenient assay for anti-Th/To and -U3RNP antibodies cannot be underestimated because these two classic anti-nucleolar autoantibodies are associated with particular SSc variants and clinical features [1]. In fact, anti-Th/To antibodies are frequently detected in lcSSc patients with pericarditis and mild lung involvement [13,20], while anti-U3RNP antibodies are associated with severe pulmonary, renal and muscular disease, mainly in African-American dcSSc patients [1,21]. Thus, the aim of our study was to establish a new method that can be used widely and easily to detect anti-Th/To and -U3RNP antibodies to replace complicated RNA analysis by gel electrophoresis.

Our results clearly show that qPCR is highly efficient and reliable to detect the RNA components of the SSc nucleolar autoantigens, when data are compared with the gold standard IP. Advantages of this new method include easy application without technically demanding RNA analysis by urea-PAGE and without the use of toxic chemicals (for example, acrylamide, silver staining) or ^32^P radioisotopes. The results obtained by our IP-qPCR assay are consistent and reproducible, and are expressed as semi-quantitative data, while the silver staining gel only detects the presence or absence of specific bands. The high sensitivity of our new method is demonstrated by requiring cell extract from only one million cells and 1 µl of serum to obtain a signal that is at least 128- to 512-fold more sensitive than standard IP-silver staining method. Another important advantage, when considering its use in a large laboratory setting, is the high number of samples that the new assay can handle at a time. In fact, a 96-well plate can be used for qPCR, while only 10 to 16 samples can be tested in a standard gel for RNA analysis by urea-PAGE.

A minor limitation of the IP-qPCR assay is the lack of information on other RNAs immunoprecipitated by the same serum sample. Examples are co-IP of 7-2 and 8-2 RNA by La antigen [18,22], and U3 RNA by rare autoimmune sera that recognize the TMG cap structure of U1-U5RNA [17]. In practice, it will be reasonable to apply this qPCR method for anti-Th/To and U3RNP antibodies only to SSc patients who are negative for anti-topo I, RNAPIII, and ACA, since the coexistence of multiple SSc autoantibodies is uncommon [1]. Also, nucleolar immunofluorescence staining in antinuclear antibody (ANA) screening will be helpful to decide which patients should be tested by this IP-qPCR method, since the majority of anti-Th/To- and U3RNP-positive patients show clear nucleolar staining [13,23], in contrast to anti-RNAPI/III sera which often do not show a nucleolar pattern [7,9]. It should be noted that the specificity of the IP-qPCR assay is nearly 100% in SSc samples and that non-SSc sera that are expected to be, or potentially are, false positives (such as anti-La, anti-TMG) were included in the study to clarify the potential limitations of the assay. The SSc sera we tested were not randomly selected, however, the proportion of each specificity is not far from a randomly selected SSc cohort, and it should give us a reasonable idea of the specificity of the assay in general SSc patients. Thus, the positive results obtained by the new IP-qPCR assay in the presence of rare antibodies in SSc, such as anti-La and anti-TMG, do not represent a clinically significant problem.

The new method can be readily applied to detect other autoantibodies, such as autoantibodies to aminoacyl-tRNA synthetases and signal recognition particle (SRP) in polymyositis/dermatomyositis patients, just by using appropriate specific primers for the RNA species of interest. With the IP-qPCR method, each tRNA specificity can be readily defined without the complicated aminoacylation assay [24]. Although clinical manifestations of many anti-synthetase antibodies appear similar and they are known as the 'anti-synthetase syndrome', it is possible that fine specificity still has clinical significance as there are some suggestions of different clinical manifestations in patients with different anti-synthetase antibodies [15,25,26].

## Conclusions

We have established a new method to detect autoantibodies to Th/To and U3RNP based on qPCR detection of the RNA components of the autoantigens. High specificity and sensitivity compared with the gold standard IP were confirmed. This new assay will be useful for detection of autoantibodies to any RNA-protein complex autoantigen and may allow clinical utilization of several autoantibodies that have been mainly detected by IP and not utilized widely in the past.

## Abbreviations

ACA: anti-centromere antibodies; ANA: anti-nuclear antibodies; cDNA: complementary DNA; Ct: cycle threshold; dcSSc: diffuse cutaneous SSc; ELISA: enzyme-linked immunosorbent assay; IP: immunoprecipitation; lcSSc: limited cutaneous SSc; NHS: normal human serum; qPCR: quantitative polymerase chain reaction; RNAPIII: RNA polymerase III; RT reverse transcription; SD: standard deviation; SRP: signal recognition particle; SSc: scleroderma: systemic sclerosis; TMG: trimethylguanosine; topo I: topoisomerase I; U3 RNP: U3 ribonucleoprotein; urea-PAGE: urea-polyacrylamide gel electrophoresis.

## Competing interests

Patent application: 'Autoantibody to RNA-protein complex detected by quantitative PCR'. U.S. PCT International Application No. PCT/US11/60789. Filing Date: November 15, 2011. Inventors: Edward K. L. Chan, Minoru Satoh, and Angela Ceribelli.

## Authors' contributions

AC and MS carried out the immunoassays. MS and EKLC designed the study. MS performed the statistical analysis. AC, MS and EKLC drafted the manuscript. All authors read and approved the final manuscript.
